# Severe influenza A in a Tunisian ICU sentinel SARI centre: Epidemiological and clinical features

**DOI:** 10.1371/journal.pone.0270814

**Published:** 2022-07-06

**Authors:** Amira Jamoussi, Samia Ayed, Takoua Merhabene, Hamdi Doghri, Jalila Ben Khelil, Mohamed Besbes

**Affiliations:** 1 University of Tunis EI Manar, Faculty of Medicine, Medical Intensive Care Unit, Abderrahmen Mami Hospital, Ariana, Tunisia; 2 Research Unit for Respiratory Failure and Mechanical Ventilation UR22SP01, Abderrahmen Mami Hospital, Ministry of Higher Education and Scientific Research, Ariana, Tunisia; Stanford University School of Medicine, UNITED STATES

## Abstract

**Introduction:**

Influenza A virus infection is a contagious acute respiratory infection which mostly evolves in an epidemic form, less frequently as pandemic outbreaks. It can take a severe clinical form that needs to be managed in intensive care unit (ICU). The aim of this study was to describe the epidemiological and clinical aspects of influenza A, then to determine independent predictive factors of ICU mortality in Abderrahmen Mami hospital, Ariana, Tunisia.

**Methods:**

It was a single-center study, including all hospitalized patients in intensive care, between November 1^st^, 2009 and October 31^st^, 2019, with influenza A virus infection. We recorded demographic, clinical and biological data, evolving features; then multivariate analysis of the predictive factors of ICU mortality was realized.

**Results:**

During the study period (10 consecutive seasons), 120 patients having severe Influenza A were admitted (Proportion = 2.5%) from all hospitalized patients, with a median age of 48 years and a gender-ratio of 1.14. Among women, 14 were pregnant. Only 7 patients (5.8%) have had seasonal flu vaccine during the year before ICU admission. The median values of the Simplified Acute Physiology Score II, Acute Physiologic and Chronic Health Evaluation II and Sepsis-related Organ Failure Assessment were respectively 26, 10 and 3. Virus strains identified with polymerase chain reaction were H_1_N_1_ pdm09 (84.2%) and H_3_N_2_ (15.8%). Antiviral therapy was prescribed in 88 (73.3%) patients. A co-infection was recorded in 19 cases: bacterial (n = 17) and aspergillaire (n = 2). An acute respiratory distress syndrome (ARDS) was diagnosed in 82 patients. Non-invasive ventilation (NIV) was conducted for 72 (60%) patients with success in 34 cases. Endotracheal intubation was performed in 59 patients with median duration of invasive mechanical ventilation 8 [3.25–13] days. The most frequent complications were acute kidney injury (n = 50, 41.7%), shock (n = 48, 40%), hospital-acquired infections (n = 46, 38.8%) and thromboembolic events (n = 19, 15.8%). The overall ICU mortality rate was of 31.7% (deceased n = 38). Independent predictive factors of ICU mortality identified were: age above 56 years (OR = 7.417; IC_95%_ [1.474–37.317]; p = 0.015), PaO_2_/FiO_2_ ≤ 95 mmHg (OR = 9.078; IC_95%_ [1.636–50.363]; p = 0.012) and lymphocytes count ≤ 1.325 10^9^/L (OR = 10.199; IC_95%_ [1.550–67.101]; p = 0.016).

**Conclusion:**

Influenza A in ICU is not uncommon, even in A(H1N1) dominant seasons; its management is highly demanding. It is responsible for considerable morbi-mortality especially in elderly patients.

## Background

According to the World Health Organization (WHO), approximately 290,000 to 650,000 deaths annually are caused by influenza virus infection alone [1]. Global influenza surveillance network was expanded to Tunisia since 1980, so surveillance and epidemiology of influenza viruses and other respiratory viruses are in progress [2–4]. Nevertheless, limited data are available about severe forms of influenza A requiring intensive care and risk factors for severe outcome in Tunisia [5].

The aims of the present study were to provide data about epidemiological and clinical features of patients admitted to the ICU with confirmed influenza A virus infection in a Tunisian referral centre and to identify independent predictors of ICU mortality.

## Methods

The study was conducted in the 22-bed medical ICU of Abderrahmen Mami Hospital in Ariana, Tunisia. This unit has a respiratory valence since it belongs to the teaching university hospital of pneumology (300 beds) above-mentioned. The mean number of ICU admissions was about 550 per year. It is also a sentinel centre among the 6 severe acute respiratory infection (SARI) surveillance centres in Tunisia [6]. It takes on surveillance of influenza from hospitalized cases under the aegis of the national programme for surveillance and control of influenza established in Tunisia in 1980 within the Department of Primary Health Care at the Ministry of Health. It involves the National Influenza Centre, recognized by the World Health Organization (WHO) in 1980 as well as a network of 268 centres for reporting influenza-like illness (ILI), which is geographically, socioeconomically and demographically representative of the Tunisian population [2].

### Study design and patients’ selection

We conducted an observational cohort study using data prospectively collected over a ten-year period (from November 1^st^, 2009 to October 31^st^, 2019). All consecutive patients aged over 16 years and diagnosed with laboratory-confirmed influenza A before or during ICU stay were included. Non-inclusion criteria were: Influenza B and age < 16 years.

Information on demographic characteristics, underlying diseases, pregnancy, seasonal flu vaccination, time to first dose of antiviral delivery, microbiological results, arterial blood gases (ABG) and biological data were recorded. Echocardiography and chest computed tomography imaging findings were collected.

Severity of the illness was evaluated according to the worst Simplified Acute Physiology Score II (SAPS II), Acute Physiology and Chronic Health Evaluation (APACHE) II score during the first 24 hours in the ICU. Organ failure was assessed using the Sequential Organ Failure Assessment (SOFA) scoring system.

Management features such as need for invasive mechanical ventilation and non-invasive mechanical ventilation, prone position, inhaled nitric oxide, catheterization, sedation, neuromuscular blockers, use of vasopressor drugs, and requirement for renal replacement therapy were also recorded. Complications occurrences during ICU stay including hospital-acquired infections, shock, acute kidney failure, bleeding, thromboembolic events and acquired neuromuscular weakness were reported. Outcome data including length of stay and ICU mortality were recorded.

### Definitions

Influenza-like illness (ILI) case definition [7] is an acute respiratory infection with:

- measured fever of ≥ 38 C°
- and cough;
- with onset within the last 10 days.

Severe acute respiratory infection (SARI) case definition [7] is an acute respiratory infection with:

- history of fever or measured fever of ≥ 38 C°;
- and cough;
- with onset within the last 10 days;
- and requires hospitalization.

Pneumonia has been considered if chest X-ray showed radiological infiltrates consistent with the diagnosis of pneumonia [8]. The distribution of radiological infiltrates was quantified in terms of affected quadrants’ number.

Acute bronchitis has been considered when cough and sputum were combined, in absence of radiological infiltrates.

Based on clinical features, imaging and PaO_2_/FiO_2_ at day 1, we classified patients according to Berlin criteria for acute respiratory distress syndrome (ARDS) [9].

Diagnosis criteria as well as stages of acute kidney injury (AKI) according to Kidney Disease Improving Global Outcomes (KDIGO) Clinical Practice Guideline for Acute Kidney Injury were adopted: an abrupt (within 48 hours) reduction in kidney function, defined as an absolute increase in serum creatinine level of 0.3 mg/dL or more, an increase in serum creatinine level of 50% or more (1.5 times as great as at baseline), or a reduction in urine output (documented oliguria of <0.5 mL/kg per hour for >6 hours) [10].

Hospital-acquired infection (HAI): infection occurred after hospital admission (≥48 h) that was neither present nor incubating on admission was considered as HAI [11].

### Laboratory testing

Samples were obtained through nasopharyngeal swab, or less often through invasive sampling (bronchial aspiration or broncho-alveolar lavage). All patients had positive result from reverse-transcriptase polymerase chain reaction tests.

### Statistical analysis

SPSS 19.0. was used for statistical analyses.

Descriptive statistics of the patients were performed and reported in terms of absolute frequencies and percentages for the qualitative variables. Quantitative variables in our cohort according to the Kolmogorov-Smirnov test were predominantly non-parametric. Quantitative variables were mostly expressed as medians and IQR 25th and 75th percentiles. Those with normal distribution were expressed in terms of means ± standard deviation (SD).

Comparisons of the clinical characteristics, biological data, complications and management requirement between survivors and non-survivors were analysed. The differences between independent groups regarding continuous variables were evaluated by Student’s t-test. Nominal data were analysed by Pearson’s Chi-square test or Fisher’s Exact test, when appropriate. Medians of quantitative variables between groups were compared using the Mann-Whitney nonparametric test.

Optimal cut off values were also determined using receiver operating characteristic curve (ROC) analysis.

Variables which showed a p value below 0.05 in the univariate analysis and results likely associated to mortality were entered into the model.

A logistic regression was performed to obtain an adjusted estimate of the odds ratios (ORs) and to identify which factors were independently associated with ICU mortality. Data were considered to be statistically significant, if the p values were less than 0.05.

### Ethical considerations

All data used in the analysis were collected in the routine public health surveillance activities, so did not require informed consent. All data were fully anonymized. This study was approved by Abderrahmen Mami Hospital’s Ethics Committee.

## Results

**Fig 1:**
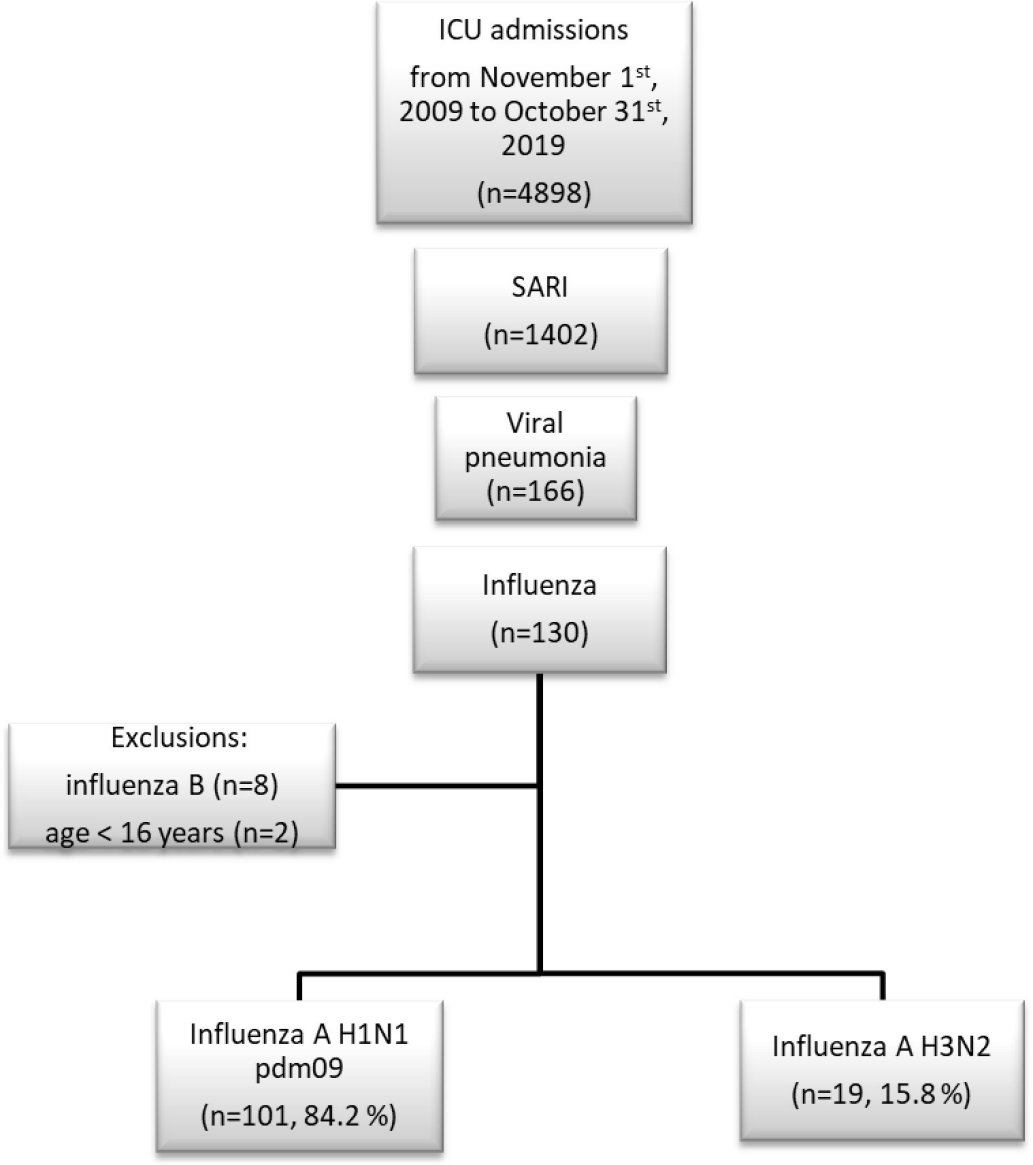
Study flowchart.

During the study period, 4898 patients were admitted to ICU from whom 120 cases of influenza A were recorded, making a proportion of 2.4% among ICU admissions (Fig 1). According to our inclusion criteria, 120 patients were finally retained to analysis, having had Influenza A, divided into 2 strains: A (H1N1) pdm09 (n = 101) and A (H3N2) (n = 19).

**Fig 2:**
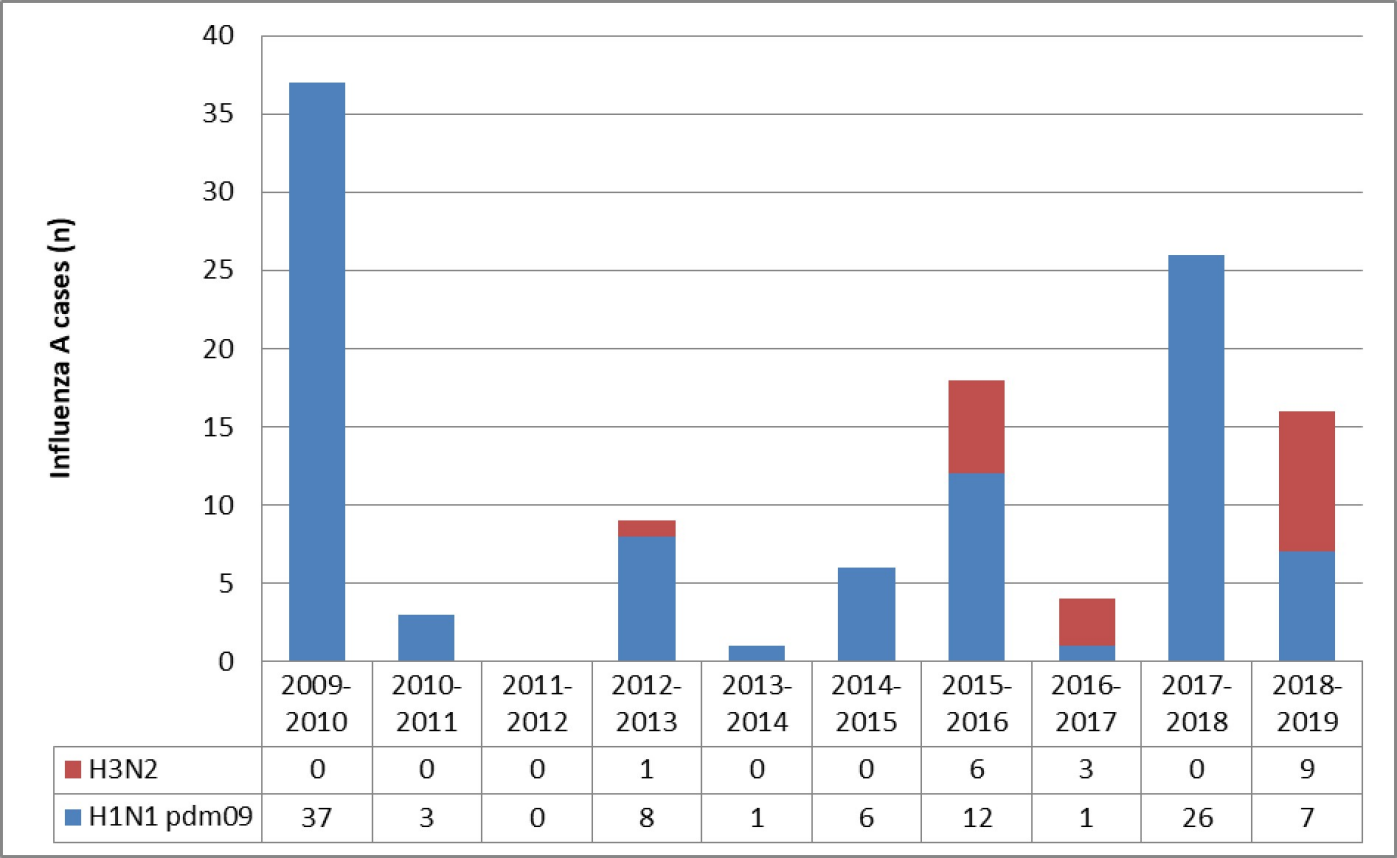
Influenza A strains distribution through 10 consecutive seasons.

**Fig 3:**
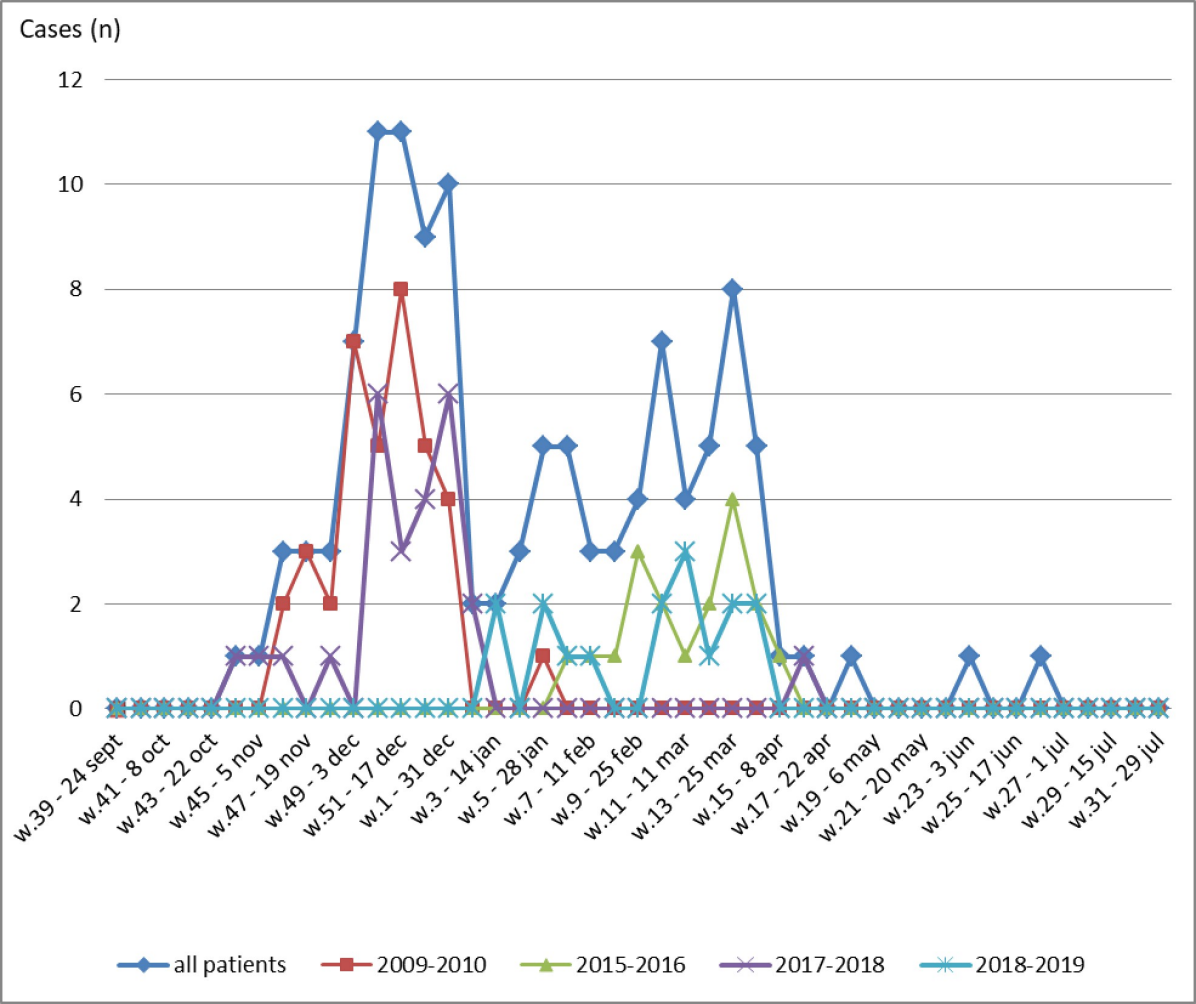
Seasonality of influenza A viruses through the 52 weeks of the year (seasons 2009–2010, 2015–2016, 2017–2018, 2018–2019 and all patients).

Influenza A frequencies and strains through seasons are illustrated in Fig 2. During these 10 consecutive seasons, peak influenza A incidence was observed mainly at 4 seasons: 2009–2010, 2015–2016, 2017–2018 and 2018–2019 (Fig 3).

### Baseline characteristics

Median age was of 48 years, IQR [37–61.7]. Forty six patients (38.3%) were aged less than 40 years. No underlying cardio-pulmonary diseases were noticed in 58 (48.3%) patients. Among enrolled patients, 55 (45.8%) were active smokers.

Gender-ratio was 1.14. Among women (n = 56), 14 were pregnant (25%). Term of pregnancy was respectively: 1^st^ trimester (n = 2), 2^nd^ trimester (n = 7) and 3^rd^ trimester (n = 5).

Pilgrimage to Mecca immediately before ICU admission was observed in 4 patients.

Patients whom have reported a contagion were 26 (21.7%). Influenza-like illness was noticed in all patients. Symptoms felt were: shortness of breath (n = 117, 97.5%), fever (n = 114, 95%), cough (n = 113, 94.2%), myalgia (n = 53, 44.2%) and arthralgia (n = 44, 36.7%). Digestive discomfort including nausea, vomiting or diarrhoea were reported in 21 patients (17.5%). Furthermore, expectorations, haemoptysis and confusion were described respectively in 79 (65.8%), 14 (11.7%) and 13 (10.8%) cases.

Prior consultations (physician’s office, clinic or emergency room) were reported in 96 patients (80%). Median number of prior consultations was 2 IQR [1–2]. Before ICU admission, the majority of patients (n = 105, 87.5%) have already taken drugs: symptomatic (anti-tussive and/or antipyretics) (n = 84, 70%), antibiotics (n = 76, 63.3%), corticosteroids (n = 31, 25.8%).

Pneumonia was present in 102 (85%) patients divided into unilateral (n = 13, 10.8%) and bilateral (n = 89, 74.2%).

An acute respiratory distress syndrome (ARDS) was diagnosed in 82 patients (68.3%) divided into severe (n = 44, 53.6%), moderate (n = 27, 33%) and mild (n = 11, 13.4%).

Baseline characteristics and onset severity of all patients are detailed in Table 1.

**Table 1:** Baseline characteristics and severity on ICU admission of 120 patients with influenza A.

	N = 120
**Age, med [IQR], years**	48 [37–61.7]
**Gender**	
Male, n (%)	64 (53.3)
Female, n (%)	56 (46.7)
**Comorbidities**	
Hypertension, n (%)	30 (25)
Diabetes, n (%)	25 (20.8)
Immunosuppression, n (%)	9 (7.5)
COPD, n (%)	15 (12.5)
Obesity, n (%)	9 (7.5)
Asthma, n (%)	11 (9.2)
**Pregnancy, n (%)**	14 (11.7)
**Seasonal flu vaccine, n (%)**	7 (5.8)
**SAPSII, med [IQR]**	26 [18–39.7]
**APACHEII, med [IQR]**	10 [6–18]
**SOFA, med [IQR]**	3 [2–6]
Reason for admission*	
Respiratory failure, n (%)	115 (95.8)
Neurologic failure, n (%)	41 (34.2)
Hemodynamic failure, n (%)	10 (8.4)
**Influenza A pneumonia,** n (%)	102 (85)
**Influenza A bronchitis,** n (%)	18 (15)
**Acute respiratory distress syndrome,** n (%)	82 (68.3)
Severe, n (%)	44 (36.7)
Moderate, n (%)	27 (22.5)
Mild, n (%)	11 (9.2)

SD: Standard Deviation; SAPS II: Simplified Acute Physiology Score II; APACHE II: Acute Physiologic and Chronic Health Evaluation II; SOFA: Sequential Organ Failure Assessment SD: standard deviation; COPD: chronic obstructive pulmonary disease;

*2 or more reasons for admission were associated in some cases.

### Paraclinical findings

Arterial blood gases at day 1 showed median pH = 7.46 IQR [7.33–7.50], median PaCO_2_ = 35 mmHg IQR [29–48], and median PaO_2_/FiO_2_ = 163 mmHg IQR [96.5–252]. At ICU admission, leucopenia (<4000/ml) was noticed in 15 patients (12.5%) and lymphopenia (< 1.500 10^9^/L) in 86 cases (71.7%). Mean blood urea nitrogen was 7.7 ± 6.2 mmol/l [1.4–37]. Rhabdomyolysis (CPK > 1000 UI/l) was almost exclusively observed with A (H1N1) pdm09 strain (n = 19, 15.8%). It was recorded in only one patient with A (H3N2) infection in a woman that had autoimmune myositis. Median CPK = 206 UI/L IQR [77.5–641] and median LDH was 488.5 UI/L IQR [315–1125].

Marked transaminases elevation was noticed in 20 patients (16.7%). Median Aspartate Aminotransferase (AST) was 46 UI/L IQR [26.5–79.5] and median Alanine Aminotransferase (ALT) was 37 UI/L IQR [24–64.2].

Transthoracic echocardiography was necessary in 92 patients (77%). It showed a preserved left ventricle systolic ejection fraction with a median of 60% IQR [50–65]. Left ventricular filling pressures were raised in 14 cases and normal in 78.

Chest X ray showed radiological infiltrates in 102 patients which was extended to 1 quadrant (n = 8, 6.7%), 2 quadrants (n = 19, 15.8%), 3 quadrants (n = 5, 4.2%) or 4 quadrants (n = 70, 58.3%).

CT scan was performed in 63 patients (52.5%) and it had shown disturbances in all cases, which most frequent were bilateral lung consolidations (n = 58) and ground glass opacities (n = 44).

Bacterial co-infection was documented in 17 (14.2%) patients: *Haemophilus influenzae* (n = 4), *Streptococcus pneumoniae* (n = 4), *Haemophilus influenzae* and *Streptococcus pneumoniae* (n = 1), *Klebsiella pneumoniae* (n = 2), *Serratia marcescens* (n = 1), *Pseudomonas aeruginosa* (n = 3), *Staphylococcus aureus* (n = 1) and *Acinetobacter baumanii* (n = 1).

Fungal co-infection with aspergillosis was documented in 2 patients. Both had bilateral pneumothorax and fatal issue.

### Management

Oseltamivir was prescribed for 88 (73.3%) patients with a delay > 48 hours for 15 patients. Daily dose was 150 mg per day (n = 52) or 300 mg per day (n = 32).

Ventilator assistance was necessary in 83 (69.2%) patients. Continuous positive airway pressure (CPAP) via Boussignac* valve was chosen for 27 patients. Only one patient was given high-flow oxygen. Non-invasive ventilation (NIV) was conducted for 72 patients with success in 34 (47.2%) cases. Endotracheal intubation was performed in 59 patients including 21 cases from the outset. Management requirements during ICU stay of all patients are detailed in Table 2.

**Table 2:** Management requirements during ICU stay of 120 ICU patients with influenza A.

	N = 120
**Oseltamivir, n(%)**	88 (73.3)
**Empirical antibiotherapy, n(%)**	106 (88.3)
**Continuous positive airway pressure, n (%)**	27 (22.5)
**Non-invasive mechanical ventilation, n (%)**	72 (60)
Success, n (%)	34 (47.2)
Failure, n (%)	38 (52.8)
**Non-invasive mechanical ventilation duration, median[IQR],** (days)	2 [1–4.5]
**Invasive mechanical ventilation, n (%)**	59 (49.2)
**Invasive mechanical ventilation duration, median[IQR],** (days)	8 [3.25–13]
**Sedation, n (%)**	49 (40.8)
**Sedation duration, median[IQR],** (days)	7 [3–8]
**Neuromuscular blockers, n (%)**	40 (33.3)
**Neuromuscular blockers duration, median[IQR],** (days)	4 [2–6]
**Prone position, n (%)**	31 (25.8)
**Prone position duration, median[IQR],** (days)	2 [1–4]
**Inhaled nitric oxide, n (%)**	12 (10)
**Inhaled nitric oxide duration, median[IQR],** (days)	1.5 [1–2.75]
**Extracorporeal membrane oxygenation, n (%)**	2 (1.7)
**Vasopressors, n (%)**	48 (40)
**Vasopressors duration, median[IQR],** (days)	4 [2–7]
**Venous catheterization, n (%)**	57 (47.5)
**Venous catheterization duration, median[IQR],** (days)	7 [5–10]
**Transfusion, n (%)**	13 (10.8)
**Renal replacement therapy, n (%)**	17 (14.2)
Intermittent	15 (12.5)
Continuous	6 (5)

### Outcomes

At least one complication occurred in 89 patients (74.2%). We recorded 75 hospital-acquired infections in 46 patients. Main complications types and frequencies are listed in Table 3.

**Table 3:** Complications among 120 ICU patients with influenza A.

	n (%)
**Bleeding**	7 (5.8)
**Thromboembolic events**	19 (15.8)
Pulmonary embolism	11 (9.2)
Deep venous thromboembolism	8 (6.7)
**Arrhythmia**	10 (8.3)
**Shock**	48 (40)
Septic	45 (37.5)
Cardiogenic	2 (1.7)
Hypovolemic	1 (0.8)
Hospital-acquired infections*	46 (38.8)
Ventilator-acquired pneumonia	31 (25.8)
Central line-associated bloodstream infection	10 (8.3)
Bacteremia	25 (20.8)
Catheter-associated Urinary Tract Infections	9 (7.5)
**Acute kidney failure**	50 (41.7)
Stage 1	20 (16.7)
Stage 2	11 (9.2)
Stage 3	19 (15.8)
**Barotrauma**	15 (12.5)
Pneumothorax	10 (8.3)
Pneumediastin	5 (4.2)
**Acquired neuromuscular weakness**	22 (18.3)
**Decubitus ulcer**	32 (26.7)

*Number of patients that had 1 or more hospital-acquired infections. CLABSI: central line-associated bloodstream infection.

### Mortality analysis

Median length of stay was of 8 days, IQR [5–13]. Overall ICU mortality was of 31.7%.

Among pregnant women, 2/14 died and 12/14 were successfully discharged. They delivered an alive baby (11/12); one woman had therapeutic abortion.

Characteristics comparison of survivors and non survivors among ICU patients with Influenza A are detailed in Table 4. Multivariate logistic regression indicated that age > 56 years, PaO_2_/FiO_2_ ≤ 95 mmHg and lymphocytes count≤ 1.325 10^9^/L at day 1 were independent risk factors for mortality (Table 5).

**Table 4:** Characteristics of survivors and non survivors among ICU patients with influenza A.

	Survivors (n = 82)	Non survivors (n = 38)	p
**Female gender, n(%)**	40 (48.7)	16 (42.1)	0.495
**Age, median[IQR], years**	42 [34.7–58.2]	59 [40.5–65]	**0.003**
**Age > 56 years, n(%)**	24 (29.2)	23 (60.5)	**0.001**
**A (H3N2) strain, n(%)**	10 (12.2)	9 (23.7)	0.109
**PaO**_**2**_ **/FiO**_**2**_ **, median[IQR], mmHg**	188 [109–262]	102 [72.5–191]	**0.001**
**PaO**_**2**_**/FiO**_**2**_ **≤ 95 mmHg, n(%)**	11 (13.4)	17 (44.7)	**<10** ^**−3**^
**Acute respiratory distress syndrome, n(%)**	46 (56.1)	36 (94.7)	**<10** ^**−3**^
**Corticosteroids prior ICU admission, n(%)**	18 (22)	13 (34.2)	0.108
**SAPS II, median[IQR]**	23 [16–33]	34 [26.7–55]	**<10** ^**−3**^
**APACHE II, median[IQR]**	8 [4.7–16.5]	15.5 [9.2–22]	**<10** ^**−3**^
**SOFA, median[IQR]**	3 [2–4.2]	5 [4–8]	**<10** ^**−3**^
**Lymphocytes count, median[IQR], 10** ^**9**^ **/L**	1 [0.7–1.525]	0.9 [0.6–1.200]	**0.047**
**Lymphocytes ≤ 1.325 10** ^**9**^ **/L, n(%)**	20 (24.4)	26 (68.4)	**0.014**
**Blood urea nitrogen, median[IQR], mmol/l**	5.1 [3.8–7.2]	8.1 [6.1–12]	**0.001**
**Blood urea nitrogen > 6 mmol/L, n(%)**	30 (36.6)	28 (73.7)	**<10** ^**−3**^
**Hepatic cytolysis n(%)**	9 (11)	11 (29)	**0.014**
**AST, median[IQR], UI/L**	46 [26–76]	44 [30–111]	0.349
**ALT, median[IQR], UI/L**	32 [22–55]	49 [27–85]	**0.041**
**ALT > 46 UI/L, n(%)**	18 (22)	22 (57.9)	**0.018**
**Bacterial coinfection, n(%)**	10 (12.2)	7 (18.4)	0.363
**Oseltamivir, n(%)**	64 (78)	24 (73.7)	0.086
**Oseltamivir ≤ 48 h, n(%)**	45 (54.9)	18 (47.4)	0.329
**Oseltamivir 150 mg/day, n(%)**	39 (47.5)	13 (34.2)	0.533
**Invasive mechanical ventilation, n(%)**	22 (26.8)	37 (97.4)	**<10** ^**−3**^
**Hospital-acquired infections, n(%)**	19 (23.2)	27 (71)	**<10** ^**−3**^
**Acute kidney failure, n(%)**	18 (22)	32 (84.2)	**<10** ^**−3**^
**Shock, n(%)**	13 (15.9)	35 (92.1)	**<10** ^**−3**^
**Acquired neuromuscular weakness, n(%)**	15 (18.3)	7 (18.4)	0.935

**Table 5:** Multivariate logistic regression.

	OR	IC 95%	p
**Age > 56 years**	7.417	1.474–37.317	**0.015**
**PaO**_**2**_**/FiO**_**2**_ **≤ 95 mmHg**	9.078	1.636–50.363	**0.012**
**Lymphocytes ≤ 1.325 10** ^**9**^ **/L**	10.199	1.550–67.101	**0.016**

## Discussion

- In this observational cohort study, we reported epidemiological data, clinical features and outcome of 120 patients with influenza A virus infection requiring ICU management in a sentinel SARI site of north Tunisia during 10 consecutive seasons from 2009 to 2019. Main findings were A(H1N1) subtype dominance, very low vaccination proportion, substantial mortality rate and independent mortality risk factors.
- Studied patients had severe influenza forms as evidenced by high severity scores at admission. ICU mortality was of 31.7%. Reported mortality from another Tunisian SARI site was about 55% [5]. Our study also shows how much influenza patients’ management in ICU is highly demanding in terms of organ replacement techniques (mechanical ventilation, renal replacement therapy, extracorporeal membrane oxygenation), explorations (CT-scan, echocardiography) and medications. This represents a heavy burden and a preventable cost to a large extent if influenza vaccination is enhanced.
- Our study revealed 3 independent predictive factors of ICU mortality: age above 56 years, deep hypoxemia as expressed with PaO_2_/FiO_2_ ≤ 95 mmHg and lymphocytes count ≤ 1.325 10^9^/L at the first ICU stay day.
- Different studies worldwide have had identified factors independently associated with mortality in patients diagnosed with influenza A and established a significant relation with very heterogeneous factors: ARDS [5], higher tidal volume [12], thrombocytopenia [13], elderly patients [14], levels of lactate dehydrogenase and white cell count on admission [15].
- The most comparable findings already published were reported in China: PaO_2_/FiO_2_ < 250 combined to lymphocyte count peripheral blood <0.83 10^9^/L considered as a simple and reliable predictor of hospitalized patients with influenza pneumonia in predicting mortality and ICU admission [16].
- Garnacho-Montero et al. focused on elderly patients with severe forms of Influenza A(H1N1)pdm09 and revealed that mortality was significantly higher in older individuals aged 65 and older [14]. Martìnez et al. investigated factors associated with death in hospitalized patients and found that the 65–74 and ≥ 75 years age groups were associated with an increased risk of death in all types and subtypes, especially for type B [17]. Age was the most frequently identified factor associated with mortality in several publications, that’s why WHO recommends seasonal flu vaccine in elderly patients over 65 years. Our study showed that the mortality risk became significantly higher from a cut-off of 56 years old. Furthermore, very few patients were vaccinated (5.8%) whereas majority of them met WHO vaccination criteria (pregnancy, chronic diseases, age). Very low vaccination incidence among studied patients may be one of the reasons that led them to have a severe influenza form needing ICU hospitalisation. This should enhance influenza vaccination of patients with risk factors because of its efficacy in preventing at least severe forms. In Tunisia, influenza vaccination policy is in accordance with the WHO recommendations. Until the 2019–2020 influenza season we used a trivalent inactivated influenza vaccine manufactured using virus grown in eggs. The quadrivalent vaccine was introduced in 2020–2021 season. Influenza vaccination is indicated for high-risk individuals: age > 65 years, chronic medical condition, immunological disorders, diabetes, obesity, healthcare workers, pregnant women, children aged between 2–5 years with chronic illness and professionals in prolonged contact with high-risk persons. Vaccination period extends from October to December every year. Despite widespread policy recommendations on influenza vaccination, coverage is low in Tunisia, through the Eastern Mediterranean Region [18] and also in most African countries [19]. Among Tunisian health professionals, there was a low vaccination rate: only 15.3% were vaccinated against influenza in the 2018–2019 influenza season [20]. To investigate reasons leading to Influenza Vaccine acceptance and decline among Tunisian Healthcare Workers, a survey was conducted including 1320 caregivers. Among them, only 43.1% were willing to receive the flu vaccine if recommended to caregivers and provided for free. Fear of the vaccine side effects was the main reason leading to vaccine decline [21].

### Patterns of seasonality

- It is known that cold temperatures facilitate the survival and spread of viruses more easily [22]. Tunisia is characterized by high humidity intervals > 60% [23]. Specific Tunisian climate parameters could explain outbreaks timings and strains identified. We mainly recorded 4 local outbreaks during the study period. We noticed that incidence peak was from week 48 to week 1 for seasons 2009–2010 and 2017–2018 where identified subtypes were A(H1N1) exclusively. However, incidence peak was recorded later from week 9 to week 15 for seasons 2015–2016 and 2018–2019 (see Fig 3).
- In this observational cohort, we registered 9 influenza A(H1N1) dominant seasons in our sentinel centre. In Tunisia, virological surveillance of Influenza viruses showed that influenza A(H1N1)pdm09 predominated over other influenza viruses (95%) in the pandemic year 2009–2010 and (70%) during 2010–11 season [2]. In 2012–2013 season, A(H1N1)pdm09 also predominated over 50.1% [4]. In 2014–2015 season, distribution of influenza viruses among positive patients was: B virus (45.3%), A(H1N1)pdm2009 (39.2%) and then A(H3N2) 15.5% [3]. According to WHO, influenza A(H3N2) activity was generally moderate. In Africa, local and regional outbreaks were reported in February and March in Egypt, Madagascar and Tunisia and during July and August in South Africa. A(H3N2) was predominant in Tunisia over 96.8% during 2013–2014 season [4].
- Through overall study period, A(H1N1) was the dominant subtype (n = 101, 84.2%) in our study. It is certainly due to epidemiological specificities reported above, but it also might result from A(H1N1) strain ability to cause severe forms. Although all strains of influenza can result in hospitalization, ICU admission or death [24], A(H1N1) virulence is well known. In a recent southern European multi‑centre cohort including 984 patients, A(H1N1) subtype was identified as an independent predictor of mortality and invasive mechanical ventilation [25].

### Antiviral therapy

- According to our national influenza surveillance guide, neuraminidase inhibitors are advocated as curative treatment in severe forms of influenza right away or getting worse [6]. Since 2007, WHO recommends Influenza antivirals since it was thought to reduce complications and transmission [26]. Neuraminidase inhibitors effectiveness is actually admitted for prevention and treatment of symptoms of influenza, but not to reduce influenza virus spread or prevention of pneumonia [27]. As already suggested by Muthuri et al., early instigation of empirical antiviral therapy should be intended as soon as possible in documented or suspected cases of influenza [28]. Nonetheless, there wasn’t significant mortality difference according to oseltamivir delay (≤48 h or after) or dose (150 vs 300 mg/day) in our study. Likewise, Rodriguez et al. showed that the administration of higher oseltamivir doses was not associated to lesser mortality [29].
- Our study has several strengths since it provides accurate data about severe influenza A patients managed in ICU in Tunisia. It highlights that influenza still may be fatal and it requires a lot of resources. The sample size is also sufficient to have significant statistics results. We also acknowledge it has limitations including single-site cohort and retrospective observational design.
- In conclusions, our study provides a comprehensive insight about the epidemiology and mortality risk factors among influenza A infected patients in a Tunisian sentinel site. These results would help in improvement and optimization of management, as well as control and prevention of severe influenza A forms. Influenza vaccination of patients with risk factors should be strongly promoted.

## Abbreviations

ICU: intensive care unit
ILI: influenza like illness
SARI: Severe acute respiratory infections
ARDS: acute respiratory distress syndrome
AKI: acute kidney failure
KDIGO: Kidney Disease Improving Global Outcomes
SAPS II: Simplified Acute Physiology Score II
APACHE II: Acute Physiologic and Chronic Health Evaluation II
SOFA: Sepsis-related Organ Failure Assessment
NIV: Non-invasive ventilation
ICD-10: International Classification of Diseases, Tenth Revision

## References

[1] Asia S. Up to 650 000 people die of respiratory diseases linked to seasonal flu each year. WHO 2017.

[2] El Moussi A, Pozo F, Kacem MABH, Ledesma J, Cuevas MT, Casas I, et al. Virological surveillance of influenza viruses during the 2008–09, 2009–10 and 2010–11 seasons in Tunisia. PloS one. 2013;8(9):e74064. doi: 10.1371/journal.pone.0074064 24069267 PMC3777972

[3] Chlif S, Aissi W, Bettaieb J, Kharroubi G, Nouira M, Yazidi R, et al. Modelling of seasonal influenza and estimation of the burden in Tunisia. EMHJ-Eastern Mediterranean Health Journal. 2016;22(7):459–66. 27714740

[4] Yazidi R, Aissi W, Bouguerra H, Nouira M, Kharroubi G, Maazaoui L, et al. Evaluation of the influenza-like illness surveillance system in Tunisia, 2012–2015. BMC public health. 2019;19(1):694. doi: 10.1186/s12889-019-7035-3 31170955 PMC6555026

[5] Bouneb R, Mellouli M, Bensoltane H, Baroudi J, Chouchene I, Boussarsar M. Characteristics and outcome of ill critical patients with influenza A infection. Pan African Medical Journal. 2018;29(1):1–8. doi: 10.11604/pamj.2018.29.174.13098 30050638 PMC6057573

[6] Guide de Surveillance de la Grippe en Tunisie 1ère Edition. 2016.

[7] OMS, Organisation Mondiale de la Santé. WHO Global Epidemiological Surveillance Standards for Influenza. 2013.

[8] Franquet T. Imaging of pneumonia: trends and algorithms. European Respiratory Journal. 2001;18(1):196–208. doi: 10.1183/09031936.01.00213501 11510793

[9] Force ADT, Ranieri V, Rubenfeld G. Acute respiratory distress syndrome. Jama. 2012;307(23):2526–33. doi: 10.1001/jama.2012.5669 22797452

[10] Kellum JA, Lameire N, Aspelin P, Barsoum RS, Burdmann EA, Goldstein SL, et al. Kidney disease: improving global outcomes (KDIGO) acute kidney injury work group. KDIGO clinical practice guideline for acute kidney injury. Kidney international supplements. 2012;2(1):1–138.

[11] System NNIS. National Nosocomial Infections Surveillance (NNIS) system report, data summary from January 1992 through June 2004, issued October 2004. Am J Infect Control. 2004;32:470–85. doi: 10.1016/S0196655304005425 15573054

[12] Oh DK, Lee MG, Choi EY, Lim J, Lee H-K, Kim SC, et al. Low–tidal volume mechanical ventilation in patients with acute respiratory distress syndrome caused by pandemic influenza A/H1N1 infection. Journal of critical care. 2013;28(4):358–64. doi: 10.1016/j.jcrc.2013.03.001 23602273

[13] Lopez-Delgado JC, Rovira A, Esteve F, Rico N, Mañez Mendiluce R, Ballús Noguera J, et al. Thrombocytopenia as a mortality risk factor in acute respiratory failure in H1N1 influenza. Swiss Med Wkly. 2013;143: w 13788. doi: 10.4414/smw.2013.13788 23739994

[14] Garnacho‐Montero J, Gutiérrez‐Pizarraya A, Márquez JA, Zaragoza R, Granada R, Ruiz‐Santana S, et al. Epidemiology, Clinical Features, and Prognosis of Elderly Adults with Severe Forms of Influenza A (H 1 N 1). J Am Geriatr Soc. 2013;61(3):350–6. doi: 10.1111/jgs.12152 23496351

[15] Hernández-Cárdenas C, Serna-Secundino H, García-Olazarán JG, Aguilar-Pérez CL, Rocha-Machado J, Campos-Calderón LF, et al. Acute respiratory distress syndrome secondary to influenza A (H1N1) pdm09: clinical characteristics and mortality predictors. Revista de Investigación Clínica. 2016;68(5):235–44. 27941959

[16] Shi SJ, Li H, Liu M, Liu YM, Zhou F, Liu B, et al. Mortality prediction to hospitalized patients with influenza pneumonia: PO2/FiO2 combined lymphocyte count is the answer. Clin Respir J. 2017;11(3):352–60. doi: 10.1111/crj.12346 26148709 PMC7162301

[17] Martínez A, Soldevila N, Romero-Tamarit A, Torner N, Godoy P, Rius C, et al. Risk factors associated with severe outcomes in adult hospitalized patients according to influenza type and subtype. PloS one. 2019;14(1):e0210353. doi: 10.1371/journal.pone.0210353 30633778 PMC6329503

[18] Abubakar A, Melhem N, Malik M, Dbaibo G, Khan WM, Zaraket H. Seasonal influenza vaccination policies in the eastern Mediterranean region: current status and the way forward. Vaccine. 2019;37(12):1601–7. doi: 10.1016/j.vaccine.2019.02.001 30795940

[19] Lagare A, Rajatonirina S, Testa J, Mamadou S. The epidemiology of seasonal influenza after the 2009 influenza pandemic in Africa: a systematic review. Afri Health Sci. 2020;20(4):1514–36. doi: 10.4314/ahs.v20i4.5 34394213 PMC8351825

[20] Cherif I, Kharroubi G, Bouabid L, Gharbi A, Boukthir A, Alaya NB, et al. Knowledge, attitudes and uptake related to influenza vaccine among healthcare workers during the 2018–2019 influenza season in Tunisia. BMC Public Health. 2021;21(1):907. doi: 10.1186/s12889-021-10970-y 33980192 PMC8116062

[21] Kharroubi G. Epidemiology and Public Health 2020: Reasons Leading to Influenza Vaccine Acceptance and Decline among Tunisian Healthcare Workers. Journal of Vaccines and Vaccination. 2020;11:1–2.

[22] Otomaru H, Kamigaki T, Tamaki R, Santo A, Daya E, Okamoto M, et al. Influenza and other respiratory viruses detected by influenza-like illness surveillance in Leyte Island, the Philippines, 2010–2013. PloS ONE. 2015;10(4):e0123755. doi: 10.1371/journal.pone.0123755 25893441 PMC4404362

[23] Brini I, Bhiri S, Ijaz M, Bouguila J, Nouri-Merchaoui S, Boughammoura L, et al. Temporal and climate characteristics of respiratory syncytial virus bronchiolitis in neonates and children in Sousse, Tunisia, during a 13-year surveillance. Environ Sci Pollut Res Int. 2020:27(19):23379–23389. doi: 10.1007/s11356-018-3922-x 30569350

[24] Lina B, Georges A, Burtseva E, Nunes MC, Andrew MK, McNeil SA, et al. Complicated hospitalization due to influenza: results from the Global Hospital Influenza Network for the 2017–2018 season. BMC Infect Dis. 2020;20(1):465. doi: 10.1186/s12879-020-05167-4 32615985 PMC7330273

[25] Almeida A, Boattini M, Christaki E, Marques TM, Moreira I, Cruz L, et al. Comparative virulence of seasonal viruses responsible for lower respiratory tract infections: a southern European multi-centre cohort study of hospital admissions. Infection. 2021;49(3):483–90. doi: 10.1007/s15010-020-01569-3 33389699 PMC7778853

[26] Organization WH. WHO interim protocol: rapid operations to contain the initial emergence of pandemic influenza. Geneva: World Health Organization. 2007.

[27] Jefferson T, Jones MA, Doshi P, Del Mar CB, Hama R, Thompson MJ, et al. Neuraminidase inhibitors for preventing and treating influenza in adults and children. Cochrane database of systematic reviews. 2014(4). doi: 10.1002/14651858.CD008965.pub4 24718923 PMC6464969

[28] Muthuri SG, Venkatesan S, Myles PR, Leonardi-Bee J, Al Khuwaitir TS, Al Mamun A, et al. Effectiveness of neuraminidase inhibitors in reducing mortality in patients admitted to hospital with influenza A H1N1pdm09 virus infection: a meta-analysis of individual participant data. Lancet Respir Med. 2014;2(5):395–404. doi: 10.1016/S2213-2600(14)70041-4 24815805 PMC6637757

[29] Rodríguez A, Díaz E, Martín-Loeches I, Sandiumenge A, Canadell L, Díaz JJ, et al. Impact of early oseltamivir treatment on outcome in critically ill patients with 2009 pandemic influenza A. J Antimicrob Chemother. 2011;66(5):1140–9. doi: 10.1093/jac/dkq511 21385717

